# The ethics of doing nothing. Suicide-bereavement and research: ethical and methodological considerations

**DOI:** 10.1017/S0033291713001670

**Published:** 2013-07-19

**Authors:** P. Omerov, G. Steineck, K. Dyregrov, B. Runeson, U. Nyberg

**Affiliations:** 1 Stockholm Centre for Psychiatric Research and Education, Department of Clinical Neuroscience, Karolinska Institutet, Sweden; 2 Division of Clinical Cancer Epidemiology, Department of Oncology–Pathology, Karolinska Institutet, Stockholm, Sweden; 3 Division of Clinical Cancer Epidemiology, Department of Oncology, Institute of Clinical Sciences, Sahlgrenska Academy, Gothenburg, Sweden; 4 The Center for Crisis Psychology, Bergen, Norway; 5 The Norwegian Institute of Public Health, Oslo, Norway

**Keywords:** Bereavement, epidemiologic methods, ethics, research design, suicide

## Abstract

**Background.:**

Valuable trauma-related research may be hindered when the risks of asking participants about traumatic events are not carefully weighed against the benefits of their participation in the research.

**Method.:**

The overall aim of our population-based survey was to improve the professional care of suicide-bereaved parents by identifying aspects of care that would be amenable to change. The study population included 666 suicide-bereaved and 377 matched (2:1) non-bereaved parents. In this article we describe the parents' perceptions of their contacts with us as well as their participation in the survey. We also present our ethical-protocol for epidemiological surveys in the aftermath of a traumatic loss.

**Results.:**

We were able to contact 1410 of the 1423 eligible parents; eight of these parents expressed resentment towards the contact. Several participants and non-participants described their psychological suffering and received help because of the contact. A total of 666 suicide-bereaved and 377 non-bereaved parents returned the questionnaire. Just two out of the 1043 answered that they might, in the long term, be negatively affected by participation in the study; one was bereaved, the other was not. A significant minority of the parents reported being temporarily negatively affected at the end of their participation, most of them referring to feelings of sadness and painful memories. In parallel, positive experiences were widely expressed and most parents found the study valuable.

**Conclusions.:**

Our findings suggest, given that the study design is ethically sound, that suicide-bereaved parents should be included in research since the benefits clearly outweigh the risks.

## Introduction

The trauma of losing a family member to suicide poses risk that survivors will experience complicated grief and long-term psychological morbidity (Kessing *et al.* 2003; Li *et al.* 2005; Groot *et al.* 2006). Adequate professional intervention might reduce this risk, but the development of evidence-based practice has been delayed by the lack of evidence-based knowledge. Institutional review boards sometimes hesitate to approve trauma-related research arguing that the contact might be hurtful and even re-traumatizing for some individuals. The risks of asking participants about traumatic events may, however, be overestimated and the benefits not considered. Therefore valuable research may be hindered (Kreicbergs *et al.* 2004*b*; Becker-Blease & Freyd, 2006; Jorm *et al.* 2007; Legerski & Bunnell, 2010).

Participants' experience of research participation has often been investigated by asking about their positive or negative emotional reactions immediately after their participation. Negative emotional reactions are often called ‘distress’ and may be described by using terms like: ‘stress, anxiety, depression, embarrassment, discomfort, negative reaction, regret of participating and intrusion of privacy’ (Jorm *et al.* 2007). Compilation of trauma-related studies suggests that a minority of participants become distressed when being interviewed or when filling out a questionnaire and that the distress quickly diminishes (Runeson & Beskow, 1991; Dyregrov *et al.* 2000; 2011; Dyregrov, 2004; Galea *et al.* 2005; Becker-Blease & Freyd, 2006; Jorm *et al.* 2007; Legerski & Bunnell, 2010). The long-term effects of participation in research have not been well studied, however (Jorm *et al.* 2007; Legerski & Bunnell, 2010). A few studies have investigated suicide-bereaved individuals' experience of research participation using a follow-up separated from the first survey or interview. Dyregrov (2004) performed a survey on psychosocial health and support among Norwegians (*n* = 262) who had lost a child to suicide, sudden infant death syndrome (SIDS) or an accident between 1997 and 1998. The survey was followed up by in-depth interviews (*n* = 69) in 1999 and a survey about the research experience, 2–4 weeks after. The survey showed that all parents (*n* = 64), even the ones who expressed the most distress and pain, evaluated their participation as positive. Runeson & Beskow (1991) explored the reactions of suicide-bereaved relatives (*n* = 58) to research participation using a structured telephone interview 2 weeks after a psychological autopsy interview and found that: 83% felt better than they had immediately after the autopsy, 57% felt better in comparison with how they had felt before the autopsy and none felt worse.

Recalling a traumatic event by telling, writing or answering questions might raise the level of short-term distress but is unlikely to cause re-traumatization or long-term harm. The temporary distress must, however, be acknowledged (Jorm *et al.* 2007; Legerski & Bunnell, 2010). Jorm *et al.* (2007) conclude that informants who might be vulnerable to distress should be treated with care but not excluded from research *per se*. Authors of several studies that included suicide-bereaved persons constructed ethical guidelines on how to reduce distress during research (Runeson & Beskow, 1991; Dyregrov, 2004; Jorm *et al.* 2007; Legerski & Bunnell, 2010). In planning our study, we considered these guidelines as well as experiences from previous studies. Despite these precautions, the regional institutional ethical committee disapproved our application with the explanation ‘great risk that a number of research participants will end up in a crisis situation or feel psychologically un-well while answering the questionnaire’. After appealing to the central institutional ethical committee, we received approval and were able to apply for our study population of suicide-bereaved and non-bereaved parents from the national registries. However, despite our ethical approval the state authorities denied our request referring to the sensitive subject and the law of secrecy. We therefore developed a new ethical protocol for epidemiological surveys of suicide-bereaved persons. In this article we present our protocol and how our study population perceived both contact with us and participation in our survey.

## Method

We developed our study design from a method that has been applied in several bereavement-related studies at the Division of Clinical Cancer Epidemiology (Kreicbergs *et al.* 2004*a*; Hauksdóttir *et al.* 2006; Rådestad *et al.* 2007; Omerov *et al.* 2013). The threats to validity were addressed by employing epidemiological methods as transferred to this field by the hierarchical step-model for study design, analysis and data interpretation (Steineck *et al.* 2006). The ethical considerations applied throughout the preparatory study and the epidemiological main study are summarized in our ethical protocol presented in Table 1.

**Table 1.** Suicide-bereaved and non-bereaved parents – a Swedish population-based survey: summary of ethical protocol for epidemiological surveys on suicide-bereaved persons

I. Preparation
Carefully plan the inclusion criteria
Same introductory letter to bereaved and non-bereaved
To meet requirements of the Swedish law of secrecy, the researchers do not receive information about the person's bereavement status. This was revealed by the informants themselves, after they had consented to being sent a questionnaire
Carefully consider when to send the introductory letter, e.g. avoiding death, name and birth dates
Make time, be prepared for long conversations with presumptive informants
Create a database for all communication and contact information
II. Introductory letter
Contact information of researchers, e.g. toll-free telephone number, availability 24 h
Focus of the study and the questionnaire
Possible negative and positive experiences of participation
Option to end participation at any time without explanation
Opportunity to decline contact or participation
Several ways to decline contact or participation, e.g. by telephone, email and text
Inform about upcoming telephone call – when and by whom
Consider and decide how many letters to send at a time in order to be able to consider and respond to informants' reactions and questions
III. Telephone call
Carefully consider when to make the telephone call, e.g. avoiding death, name and birth dates
Telephone call by trained interviewer
Careful sensitive ‘step-by-step’ approach going from general questions to more detailed ones
Being responsive and prepared for questions and need of support
Provide support and help with referral if needed
Encourage contact again if help or support is needed
Give enough time for questions and support
Accept a denial directly without further probing
Repeat option to end participation at any time without explanation
Ask for consent to send a questionnaire
Ask for consent to call again within a time agreed upon
IV. During participation
Continuity throughout the study with the same trained interviewers
Interviewers being available and prepared for questions and support 24 h
Provide support and help with referral if needed
Give enough time for questions and support
Give enough time for participation, e.g. being able to return questionnaire within a wide time-frame
Ask for consent to call again during participation

### Preparatory study

The overall aim of our study was to improve the professional care of suicide-bereaved parents by identifying aspects of care that could be amenable to change. We created our hypotheses and a questionnaire in our pre-study that included: a literature review, in-depth interviews with 17 suicide-bereaved parents, qualitative content analysis, validation of the questionnaire and a pilot study. The validation process involved 46 suicide-bereaved parents and external experts such as other researchers and clinicians (Omerov *et al.* 2013). Along with ensuring the validity of the questions we also strove to phrase the questions as inoffensively as possible. The questionnaire for the suicide-bereaved parents contained 196 main questions with follow-up questions and fields for free comments covering the time before death, the death and the time after the death. Our primary outcome of depression was measured by the nine-item depression scale of the Patient Health Questionnaire (PHQ-9) (Kroenke *et al.* 2001; Omerov *et al.* 2013). Symptoms of anxiety and depression were also assessed by study-specific questions based on the Diagnostic and Statistical Manual of Mental Disorders, Fourth Edition (DSM-IV) criteria (APA, 1995; Omerov *et al.* 2013). Since depression and anxiety are common in the general population we included a matched control group of non-bereaved parents for comparison. The non-bereaved parents received a shortened version of the questionnaire that had 93 questions, follow-up questions and fields for free comments. In this questionnaire we included the questions that address the parents' well-being and daily life, as well as the psychometric scales. The questions regarding research participation presented in Table 2 were developed from similar questions in previous research (Dyregrov, 2004; Kreicbergs *et al.* 2004*b*).

**Table 2.** Participation and characteristics of suicide bereaved and non-bereaved parents

	Suicide-bereaved^a^	Non-bereaved^b^
Eligible parents, *n* (%)	915 (100)	508 (100)
Fathers	424 (46)	232 (46)
Mothers	491 (54)	276 (54)
Did not provide information, *n* (%)	249 (27)	131 (26)
Not reachable	8 (<1)	5 (1)
Declined participation	125 (14)	88 (17)
Agreed but did not participate	116 (13)	38 (7)
Provided information, *n* (%)	666 (73)	377 (74)
Fathers	283 (67)	166 (72)
Mothers	383 (78)	211 (76)
Characteristics of participating parents, *n*	666	377
Gender, *n* (%)		
Fathers	283 (42)	166 (44)
Mothers	383 (58)	211 (56)
Median age, years (interquartile range)		
Fathers	58 (53–62)	59 (54–62)
Mothers	55 (51–59)	54 (50–59)
Year of child's death, *n* (%)		Not applicable
2004	162 (24)	
2005	174 (26)	
2006	169 (25)	
2007	161 (24)	
Median age of deceased child, years (interquartile range)	23 (20–27)	Not applicable
Gender of deceased child, *n* (%)		Not applicable
Male	462 (69)	
Female	204 (31)	
Children, *n* (%)^c^		
One child	71 (11)	43 (11)
Two children	241 (36)	139 (37)
Three or more children	350 (53)	193 (51)
Not stated	4 (<1)	2 (<1)
Biological children	635 (95)	369 (98)
Non-biological children	31 (5)	7 (2)
Not stated	0 (0)	1 (<1)
Family constellation at time of study, *n* (%)		
Living with a partner	477 (72)	271 (72)
Has a partner but lives alone	44 (7)	28 (7)
Single	121 (18)	67 (18)
Widow, widower	18 (3)	11 (3)
Not stated	6 (<1)	0 (0)
Residence area, *n* (%)		
Rural	162 (24)	77 (20)
Village (population <10 000)	153 (23)	97 (26)
Small town (population <50 000)	128 (19)	73 (19)
Town (population <200 000)	117 (18)	62 (16)
Larger town (population >200 000)	97 (15)	68 (18)
Not stated	9 (1)	0 (0)
Country of birth, *n* (%)		
Born in Sweden	629 (94)	371 (98)
Born in other Nordic country	36 (6)	6 (2)
Not stated	1 (<1)	0 (0)
Level of education, *n* (%)		
Less than primary school	5 (<1)	2 (<1)
Primary school	141 (21)	71 (19)
Secondary school	271 (41)	158 (42)
Higher education (<3 years)	82 (12)	55 (15)
Higher education (>3 years)	159 (24)	91 (24)
Not stated	8 (1)	0 (0)
Source of income, *n* (%)		
Employed or self-employed	498 (75)	303 (80)
Old-age pension	59 (9)	38 (10)
Disability pension	61 (9)	21 (6)
Unemployment fund	25 (4)	6 (2)
Study allowance	4 (1)	0 (0)
Social security	3 (0)	0 (0)
Other	9 (1)	9 (2)
Not stated	7 (1)	0 (0)
Annual income in Swedish crowns, *n* (%)		
0–99 000	34 (5)	10 (3)
100 000–199 000	120 (18)	64 (17)
200 000–399 000	388 (58)	240 (64)
400 000 or more	109 (16)	59 (16)
Not stated	15 (2)	4 (1)
Religion, *n* (%)		
Do not believe in God	355 (53)	216 (57)
Believes in God	287 (43)	150 (40)
Not stated	24 (4)	11 (3)

a Parents who, according to the registers, had lost a son or daughter to suicide, age 15–30 years, between 2004 and 2007. Parents born outside a Nordic country, without a registered address and telephone number, who could not speak Swedish, or had lost more than one child were excluded.

b Non-bereaved parents matched for gender, age, marital status, index child, number of children and residence area. The inclusion criteria were identical, except that they were not allowed to have lost a child.

c The suicide-bereaved parents' dead child is included in the figures.

### Epidemiological main study

The parents that had lost a 15- to 30-year-old son or daughter through suicide 2–5 years earlier were identified by linkage of the Swedish Cause of Death Register and the Multi-generation Register. To be included in the study, the parent had to be born in one of the Nordic countries, be able to communicate in Swedish and have an identifiable address and telephone number. Furthermore, parents that had lost more than one child were excluded. A random sample of non-bereaved parents matched for age, gender, living area, marital status, number of children, and with a child born the same year as the deceased child was identified through the Swedish Population Register. To keep our procedures within the bounds specified by the Swedish ‘law of secrecy’, the identification of the suicide-bereaved and the matching of non-bereaved parents were done by the register holders and the researchers did not know whether the parents were bereaved or non-bereaved until they chose to reveal this themselves. We contacted all parents by means of an introductory letter followed by a telephone call after 2 weeks (Beskow *et al.* 1990; Dyregrov, 2004; Jorm *et al.* 2007; Eilegård *et al.* 2013). To minimize the risk of upsetting parents who were uncertain about the cause of death or believed that the cause of death was something other than suicide, we only included deaths registered as suicides [International Classification of Diseases, Tenth Revision (ICD-10) code X60–84] and did not include deaths for which the cause was uncertain (ICD-10 code Y10–34). Although this may appear to be unusual, we had to consider the possibility that a few of the deaths had been erroneously classified as suicide. In the introductory letter we therefore addressed the parents as ‘someone who has lost a son or daughter in a sudden death’ and someone ‘who has not lost a son or daughter’ and emphasized that the questionnaires were developed together with suicide-bereaved parents. We also forewarned participants that some of the questions could raise difficult emotions, although participants in similar studies often perceived the participation as valuable (Dyregrov, 2004; Jorm *et al.* 2007; Dyregrov *et al.* 2010). In the introductory letter we emphasized that participation was voluntary and informed about the possibility to end participation at any time without further explanation (Jorm *et al.* 2007). The researchers' names and telephone numbers, one of which was toll free, were listed and the parents were encouraged to contact us with questions or if they needed support at any time during the study. For ethical reasons we made it easy to decline without any need for personal contact by indicating that they could decline by means of email, letter, text message or by leaving a message on an answering machine. We only sent around 50–100 introductory letters each week since we wanted to have time to attend to incoming and follow-up telephone calls. This time-frame also enabled us to stop the data collection if our research were to prove to be harmful to the participants in any way. During the whole study we avoided contact on official holidays and weeks containing birthdays, name days and the date of the death (fictional for the non-bereaved). We used a tailor-made database to enable a safe and systematic data collection. All events as well as the parents' comments were carefully noted and registered in the database.

The telephone calls were made by an experienced research assistant or by the first author (P.O.) who is a registered nurse specialized in psychiatry (Omerov *et al.* 2013). To avoid distress and personal intrusion, all calls were made using a sensitive ‘step-by-step approach’, meaning that we started with general questions and were responsive to any indication that it was time to stop probing. A denial was accepted immediately without challenging the decision or trying to persuade the parent to participate. Spontaneous motivations for the denials were noted and sorted according to categories established in the pilot study (Omerov *et al.* 2013). Usually, we started the telephone conversation by asking the parent if he or she had read the introductory letter and whether the parent had any questions. If the informant did not decline or did agree to participate directly, which was the common case, we asked if he or she wanted to look at a questionnaire. If the answer was yes, we then asked if he or she had lost a son or daughter. If this was the case, we explained that the questionnaire had been developed in cooperation with suicide-bereaved parents, which often resulted in a comment about their own son or daughter's cause of death. A few parents told us that the cause of death was unknown to them or that their son or daughter had died in an accident or had been murdered. The callers were always prepared to listen for as long a time as was needed (Dyregrov, 2004). All parents that expressed a need for support were offered the chance to talk with the first author who has long experience working with traumatized patients and suicide-related issues. A few parents needed further professional intervention and were either aided in obtaining appropriate help or were offered the chance to speak with the last author (U.N.) who is a physician specialized in psychiatry as well as suicidology. We emphasized that participation could be ended at any time without further explanation and also informed the parent about the possibility to answer the questionnaire anonymously. At each telephone call we asked for consent to call again if the parent had not returned the questionnaire within a time-frame that we had agreed upon (Omerov *et al.* 2013).

## Results

### Participants

The questionnaires were returned by 666 of the 915 (73%) suicide-bereaved, and 377 of the 508 (74%) non-bereaved parents (Table 2). The mean length of time for completing the questionnaire was 38 days (median 19 days). The mean answering rate for the main questions was 98% (data not shown in the table). A majority, 633 of 666 (95%) bereaved and 347 of 377 (92%) non-bereaved parents, answered that they thought that the study was valuable, and 604 of 666 (91%) and 287 of 377 (76%) that they would recommend another parent to participate. We found that 334 of 666 (50%) and 104 of 377 (28%) reported being positively affected by their participation, whereas 70 of 666 (11%) and three of 377 (1%) reported being negatively affected (Table 3). Of the suicide-bereaved that reported being negatively affected, 51 referred to painful memories in their written comments and 10 wrote that they felt sad or depressed. Some commented that these feelings were not necessarily bad for them and 36 of 70 (51%) reported being both negatively and positively affected by their participation (data not shown in the table). Among the suicide-bereaved parents that reported being negatively affected, 14 of 69 (20%) were moderately to severely depressed (score 10 or more) according to the PHQ-9. In total, 115 of 665 (18%) suicide-bereaved and 28 of 374 (8%) non-bereaved parents were moderately to severely depressed (score 10 or more) according to the PHQ-9 (data not shown in the table). Only one suicide-bereaved and one non-bereaved parent answered that they thought that the negative effect might last. In all, 25 of 666 (4%) bereaved and 17 of 377 (5%) non-bereaved parents answered that they regretted their participation. Of the 25 suicide-bereaved, 14 commented on this answer; eight referred to painful memories and sadness; five to too many questions and one parent perceived the questionnaire as impersonal. Of the 17 non-bereaved parents, four commented on their answers; one referred to ongoing cancer disease, one that she had not lost a child, one to research participation in general and one to low mood. A total of 265 of the suicide-bereaved parents commented in response to the question on being positively affected by participation. The comments fit predominantly into three categories: (i) gratitude for the opportunity to relate experiences and for interest in the child, situation and subject; (ii) hope that relating their experiences might help others in a similar situation and improve care provision; and (iii) experience of being helped by working through memories and feelings raised by answering the questionnaire. The non-bereaved parents wrote 78 comments in reply to the question and most of them referred to gratefulness for helping others and gratefulness for having their child and their health.

**Table 3.** Experience of research participation

	Suicide-bereaved parents	Non-bereaved parents
Do you think it's valuable to conduct such a survey?		
No	8/666 (1.2)	12/377 (3.2)
Yes	633/666 (95.0)	347/377 (92.0)
Yes, a little	38/666 (5.7)	60/377 (15.9)
Yes, rather much	112/666 (16.8)	135/377 (35.8)
Yes, very much	483/666 (72.5)	152/377 (40.2)
Not stated	25/666 (3.8)	18/377 (4.8)
Do you think this survey has had a negative effect on you?		
No	574/666 (86.2)	363/377 (96.3)
Yes	70/666 (10.5)	3/377 (0.8)
Not stated	22/666 (3.3)	11/377 (2.9)
If yes, do you think this negative effect will last?		
No	65/70 (92.9)	2/3 (66.7)
Yes	1/70 (1.4)	1/3 (33.3)
Not stated	4/70 (5.7)	0/3 (0)
Do you think this survey has had a positive effect on you?		
No	293/666 (44.0)	256/377 (67.9)
Yes	334/666 (50.2)	104/377 (27.6)
Not stated	39/666 (5.9)	17/377 (4.5)
If yes, do you think this positive effect will last?		
No	77/334 (23.0)	34/104 (32.7)
Yes	198/334 (59.3)	55/104 (52.9)
Not stated	59/334 (17.7)	15/104 (14.4)
Would you recommend another parent to participate in this study?		
No	37/666 (5.6)	72/377 (19.1)
Yes	604/666 (90.7)	287/377 (76.1)
Yes, a little	102/666 (15.3)	96/377(25.5)
Yes, rather much	166/666 (24.9)	95/377 (25.2)
Yes, very much	336/666 (50.5)	96/377 (25.5)
Not stated	25/666 (3.8)	18/377 (4.8)
Do you regret participating in this study?		
No	635/666 (95.3)	349/377 (92.6)
Yes	25/666 (3.8)	17/377 (4.5)
Yes, a little	20/666 (3.0)	10/377 (2.7)
Yes, rather much	4/666 (0.6)	0/377 (0)
Yes, very much	1/666 (0.2)	7/377 (1.9)
Not stated	6/666 (0.9)	11/377 (2.9)

Data are given as number of participants/total number of participants (percentage).

### Non-participants

**Fig. 1.**
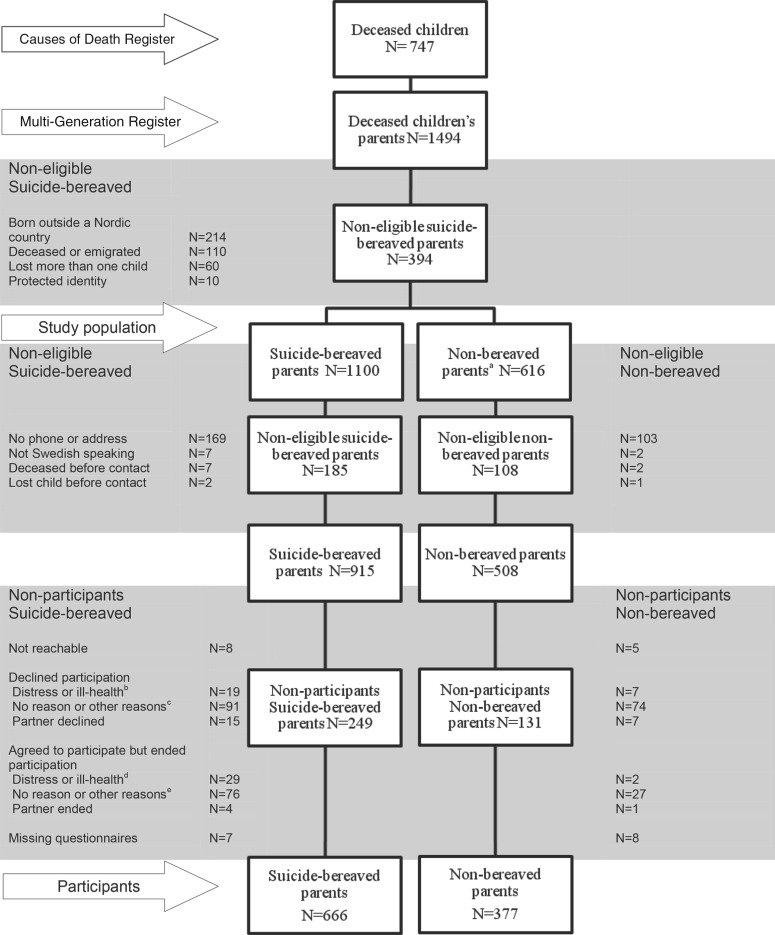
Participation and non-participation among suicide-bereaved and non-bereaved parents. All information in this figure is based on information from the registries (only group level) or from the parents themselves. Due to the requirements of the Swedish ‘act of secrecy’ the researchers did not know if the parent was bereaved or non-bereaved until he or she chose to reveal this personally. ^a^ The non-bereaved parents were matched with the suicide-bereaved parents in a ratio of 2:1 on the following variables: marital status, age, gender, living area and number of children. All the non-bereaved participants had a child born the same year as the deceased child's age. All fulfilled the same inclusion criteria as the suicide-bereaved parents: was born in a Nordic country and had a listed telephone number and address. ^b^ A total of 26 parents declined due to psychological distress or ill-health and four to somatic disease or conditions. The same reasons could be found among the partners who declined. ^c^ Other reasons were mainly related to unwillingness to participate in research *per se* (*n* = 22), ‘lack of time’ (*n* = 7) or the cause of death being something other than suicide (*n* = 6). ^d^ In all, 31 parents ended participation due to psychological distress or ill-health and two to a somatic disease or conditions. The same reasons could be found among the partners who declined. ^e^ Around 50 parents ended their participation without given reasons and about 50 referred to ‘lack of time’ or a ‘complicated life situation’.

A total of 13 parents could not be reached by telephone or email; eight of them were suicide-bereaved and five non-bereaved, according to the registries (Fig. 1). Most of the 213 parents that declined participation did so in a friendly manner without hesitation (data not shown in the table). Of those who spontaneously gave a motivation to their decision not to participate, 26 were categorized as due to ‘distress or ill-health’ of which 22 referred to ongoing psychological distress or ill-health and four to somatic diseases or conditions. Similar reasons were given for the 22 cases in which participation was declined by another person. In all, 21 parents said that they did not participate in research as a principle and seven persons referred to ‘lack of time’. Of those who did not want to participate, six persons stated that their son or daughter had died from causes other than suicide (Fig. 1). Several of the parents that declined regarded the research group as connected to the healthcare system and expressed disappointment over the health care that the child had received. Professional encounters, both before and after the suicide, that were perceived as hurtful were also commonly described.

### Offended non-participants

In total, we concluded that eight parents had been offended by the contact *per se*. Of these, six persons denied participation by expressing irritation or anger; two persons were initially shocked and distressed that the cause of death had been attributed to suicide; one person wanted our help to reinvestigate the cause of death and later expressed gratitude over the help he received. The other person accepted our offer of a follow-up call but later chose to communicate that she felt better through her spouse. The spouse, who chose to participate in the study, said that despite her negative reaction, he believed that the contact had had a positive overall effect on their family.

### Agreed to participate but ended participation

In total, 31 of the bereaved parents agreed to participate and received a questionnaire but ended their participation due to ‘distress or ill-health’; two of them referred to somatic diseases and the rest to psychological distress or ill-health that had started before the contact with us (Fig. 1). Around half of these parents received support over the telephone with the first author, and a handful were also referred to the last author. Several had ongoing contact with other health professionals and others were supported in finding a suitable contact. A few participants with an ongoing depression or an anxiety disorder were encouraged to end their participation after describing how they had struggled but failed to answer the questions. Around 50 parents ended their participation without giving any explanation and around 50 referred to ‘lack of time’ or a ‘complicated life situation’. Some parents thought that the questionnaire was too extensive and a few that it did not match their special circumstances. A few participants or relatives to the participants perceived the questions as being too personal. Some of the parents (15 in all) claimed to have returned their questionnaires, but these could not be found (Fig. 1).

## Discussion

We investigated how 666 suicide-bereaved and 377 non-bereaved parents perceived their research participation in an extensive population-based survey. A minority of the participants answered that they were negatively affected by the participation and in total two out of 1043 stated that they thought the negative effects might last; one was bereaved, the other was not. On the other hand, positive experiences were widely expressed and 94% of the parents thought that the study was valuable.

Our findings correspond with Eilegård *et al.* (2013) who found that none of 168 bereaved siblings thought that their research participation would affect them negatively in the long term. The siblings had lost a brother or sister to cancer and answered a survey 2–9 years after their loss. This population-based survey was based on the same methodology as our survey and used identical questions to measure long-term experience of research participation. As in previous studies, only a minority of the participants reported being negatively affected at the time of participation and compilation of the findings from previous studies suggests that negative feelings rapidly decrease with time (Runeson & Beskow, 1991; Kreicbergs *et al.* 2004*b*; Jorm *et al.* 2007; Legerski & Bunnell, 2010). However, a significant group of parents, especially among the suicide-bereaved, agreed to participate but then withdrew, several referring to distress or ill-health. It is our impression that the motivation to participate was stronger among the suicide-bereaved parents in comparison with the non-bereaved. At the same time, psychological morbidity prior to participation was higher among the suicide-bereaved who also received a longer and more emotionally-challenging questionnaire. We spoke to nearly every one of the suicide-bereaved that withdrew their participation and no one expressed that they regretted their initial consent to participate. Although no one concluded that their distress was caused by their participation, several parents said that answering the questions was too much of a struggle in their present state of psychological ill-health. One may hypothesize that if these parents had fulfilled their participation, the percentage of negatively affected persons would have been higher. We also found that some parents that were eager to continue despite severe distress needed encouragement to end their participation, which emphasizes the importance of having a personal contact.

As in previous investigations, the majority of our participants found their participation valuable (Runeson & Beskow, 1991; Dyregrov, 2004; Kreicbergs *et al.* 2004*b*; Jorm *et al.* 2007; Legerski & Bunnell, 2010; Eilegård *et al.* 2013). Several parents expressed that the personal telephone contact was valuable and welcomed the opportunity to tell about their experiences. The opportunity to disseminate knowledge about their situation was also emphasized as positive in their written comments. The suicide-bereaved parents also wrote that going through the questions was helpful since it aided them to remember and work through emotions. At the same time, ‘painful memories’ and ‘feelings of sadness’ were the most common motivations to being negatively affected or regretting participation. This shows the value of letting the respondents themselves rate if they were negatively affected or not since the respondents are the only ones that can put this question in a context of their whole situation. The wording ‘negative or positive’ might, however, be misleading since immediate undesirable reactions may be beneficial in the long term, a possibility that was also suggested by the participants themselves (Dyregrov, 2004; Jorm *et al.* 2007; Dyregrov *et al.* 2010). Some parents also referred to practical issues when they rated being ‘negatively affected’ and if they regretted their research participation, which shows that different evaluation criteria were used when answering the questions. Our control group consisted of matched parents who had not lost a child. Their shortened questionnaires involved a few questions regarding their experience of death and suicide; otherwise they were not asked to recall any traumatic events. The prevalence of negatively affected parents in the control population suggests that some of the effects cannot be explained by answering questions about a specific trauma, which is also supported by previous research (Jorm *et al.* 2007).

Our impression was that most parents did not have a problem with either declining or accepting participation, a finding supported by previous studies (Jorm *et al.* 2007; Dyregrov *et al.* 2011). The introduction letter gave the presumptive participant time to consider and to prepare his or her decision before the telephone call. We also made it easy to decline participation without personal contact. It is sometimes argued that it is more ethical to leave it up to the presumptive participant to initiate the contact after receiving the information letter, but this dramatically decreases the response rate (Beskow *et al.* 1990; Eilegård *et al.* 2013). Also, a personal telephone call enables the researchers to respond to reactions to the contact. In our study, eight of 1423 parents expressed distress related to the contact. In two cases the distress was later transformed into gratitude for the help received. We believe that our study design that thoroughly considered every detail in the written and personal contact reduced the number of distressed persons. However, we do not know anything about the 13 parents that could not be reached. One may hypothesize that the number of parents being initially distressed would have been higher if we had included the causes of death that were registered as uncertain, although we know that most of these are suicides. It is also possible that some of the ones that chose not to participate without giving an explanation were distressed about the contact. It is important to note that our contact provided essential and sometimes crucial help for several non-participants and participants that suffered from psychological distress or ill-health.

Our study has several strengths: one is the large sample of suicide-bereaved parents and matched controls, all identified through nationwide high-quality registers (Ludvigsson *et al.* 2009); another is the high participation rate (Table 2). We addressed the threats to validity by the hierarchical step-model (Steineck *et al.* 2006). First, we had to consider possible confounding factors. We did this by matching the suicide-bereaved with the non-bereaved parents on sociodemographic variables and by measuring other possible confounding factors in the questionnaire (Omerov *et al.* 2013). We found that the factors we matched for as well as the measured ones showed high concordance among the groups of respondents (Table 2). Second, we used several measures to reach a sufficiently high participation rate. In total, 73% parents answered our questionnaire, which should be high enough to avoid systematic errors related to misrepresentation. Third, in order to reduce the risk of misclassification we tested all questions in our thorough preparatory study with parents from our study population (Charlton, 2000; Edwards *et al.* 2009; Alderman & Salem, 2010; Omerov *et al.* 2013). Our study also has limitations. In all, 26% of the eligible parents did not answer the questionnaire and we do not know how they would have answered the questions of interest and whether their participation would have affected our findings. We know that several of the suicide-bereaved decided not to participate because of psychological distress or morbidity and that some declined participation to avoid additional distress. One may hypothesize that this group would be more affected by the participation which would lead to an underestimation of the ones reporting being both negatively and positively affected by the participation (Legerski & Bunnell, 2010). We made the choice not to collect longitudinal data, since previous studies show that it is difficult to maintain sufficient response rates in these kind of surveys (Clark, 2001). This, and the opportunity of answering anonymously, disabled us from measuring the participants' actual long-term experience of research participation. Instead, we had to ask the participants themselves if they thought that any negative or positive effect of their participation would last. Only a minority reported being negatively affected by the research participation and previous studies suggest that it is unlikely that persons that were not negatively affected initially would be that later on as a consequence of research participation (Legerski & Bunnell, 2010). The primary manifestation of research participation and distress might be universal; still, generalizability to other suicide-bereaved populations may be compromised by culture-specific issues.

## Conclusions

Our findings suggest, given that the study design is ethically and methodologically sound, that suicide-bereaved parents should be included in research since the benefits clearly outnumber the risks. Almost all parents found the study valuable and the need for the research was strongly emphasized. Also of utmost importance, several non-participants and participants described severe psychological suffering and received help because of the contact. The high prevalence of depression among the bereaved suggests that professional interventions might be useful to reduce psychological morbidity. However, evidence to guide these interventions is sparse and more research is needed. This conclusion has to be weighed against the finding that a few parents did express distress related to the contact and participation.
